# Epigenetic Regulation of Anthocyanin Biosynthesis in *Betula pendula* ‘Purple Rain’

**DOI:** 10.3390/ijms252212030

**Published:** 2024-11-08

**Authors:** Chenrui Gu, Huan Xu, Qihang Yuan, Jinbo Huang, Kunying Yuan, Yihan Zhao, Guifeng Liu, Qingzhu Zhang, Jing Jiang

**Affiliations:** 1 State Key Laboratory of Tree Genetics and Breeding, Chinese Academy of Forestry, Northeast Forestry University, Harbin 150040, China; guchenrui@outlook.com (C.G.); xuhuan157@163.com (H.X.); yuansail@foxmail.com (Q.Y.); 18856039122@163.com (J.H.); yuankunying0131@163.com (K.Y.); zhaoyihan0028@163.com (Y.Z.); liuguifeng@126.com (G.L.); 2 College of Life Science, Northeast Forestry University, Harbin 150040, China

**Keywords:** *Betula pendula* ‘Purple Rain’, DNA methylation, MYB, anthocyanin biosynthesis

## Abstract

*Betula pendula* ‘Purple Rain’ is characterized by its purple leaves and has ornamental applications. A green mutant line NL, which was mutated by line NZ of *B. pendula* ‘Purple Rain’ during tissue culture, shows green leaves instead of the typical purple color of *B. pendula* ‘Purple Rain’. This study quantified the leaf color traits of NL and a normal *B. pendula* ‘Purple Rain’ line NZ, and uncovered differentially expressed genes involved in flavonoid biosynthesis pathway genes in NL through RNA-Seq analysis. Compared to NZ, reduced levels of six anthocyanins contained in NL were revealed via flavonoids-targeted metabolomics. Sequence mutations in transcription factors that could explain NL’s phenotype failed to be screened via whole-genome resequencing, suggesting an epigenetic basis for this variant. Therefore, a key gene, *BpMYB113*, was identified in NL via the combined analysis of small RNA sequencing, whole-genome methylation sequencing, and transcriptomics. In NL, this gene features a hyper CHH context methylation site and a lower transcription level compared to NZ, disrupting the expression of downstream genes in the phenylalanine metabolism pathway, and thereby reducing flavonoid biosynthesis. Our study elucidates an epigenetic mechanism underlying color variation in variegated trees, providing pivotal insights for the breeding and propagation of colored-leaf tree species.

## 1. Introduction

Ornamental foliage plants, characterized by their prolonged aesthetic appeal, high color intensity, and diverse coloration, have become integral components in contemporary landscape architecture. Leaf coloration primarily results from variations in the proportions of chlorophyll, carotenoids, and flavonoids, with flavonoids playing a dominant role. Flavonoids constitute a broad class of compounds unified by a common C6-C3-C6 structure, formed by two benzene rings linked through three carbon atoms, exhibiting extensive diversity and commonly classified into anthocyanins, proanthocyanins, flavonols, isoflavones, flavanones, and flavones [1,2]. Flavonoid compounds possess functionalities to mitigate both biotic and abiotic stresses, and they also hold significant positions in medicine and pharmaceuticals, and as nutritional compounds [3]. Anthocyanins are the predominant flavonoid pigments responsible for the reddish or purple hues observed in plant tissues. The anthocyanin biosynthetic pathway, conserved among higher plants, initiates with cytoplasmic phenylalanine and proceeds through a series of enzymatic reactions catalyzed by phenylalanine ammonia-lyase (PAL), cinnamate 4-hydroxylase (C4H), 4-coumarate-CoA ligase (4CL), chalcone synthase (CHS), chalcone isomerase (CHI), flavonoid 3′-hydroxylase (F3′H), flavanone 3-hydroxylase (F3H), dihydroflavonol 4-reductase (DFR), and anthocyanidin synthase (ANS) [4,5,6]. The levels of these flavonoids and their intermediates in the biosynthetic pathway can be quantified using metabolomics techniques, such as ultra-performance liquid chromatography coupled with tandem mass spectrometry (UPLC-MS/MS), which offers high sensitivity and resolution. Integrating these metabolic profiling outcomes with transcriptomic sequencing data facilitates an in-depth investigation into the variations occurring at different stages of the anthocyanin biosynthesis pathway [2,7,8].

Anthocyanin biosynthesis is predominantly regulated by three classes of transcription factors, *MYB*, *bHLH*, and *WDR*, which together form the MBW complex [9]. The sequences of the MYB domain are highly conserved. Gene subfamilies containing two to three repeats of this conserved MYB motif are designated as *R2R3-MYB*. Within the MBW complex, *R2R3-MYB* family proteins play a principal role, typically exerting positive control over anthocyanin synthesis [10,11,12,13]. *MYB113* and *MYB114* share sequence similarities and belong to the *R2R3-MYB* subfamily [14]. These MYB proteins positively modulate anthocyanin synthesis [15], functioning as key inducers of anthocyanin production in trees, exemplified by *EgrMYB113* in *Eucalyptus grandis* [16], *XsMYB113-1* in *Xanthoceras sorbifolium* [17], the MYB113-like transcription factor *GbBM* in *Gossypium hirsutum* [18], and *MdMYB114* in *Malus domestica* [19]. Furthermore, several additional transcription factors, including *COP1* [20,21], *JAZ* [20], *NAC* [22], *SPL* [23], and *WRKY* [24], can influence anthocyanin biosynthesis through interactions with the MBW complex, thereby exerting secondary regulatory effects on anthocyanin content in plants.

DNA methylation in plants constitutes a chemical modification (^5m^C) of genomic cytosines, capable of altering gene expression without modifying sequences. It is widely regarded that DNA methylation suppresses gene expression [25]. This suppression arises from the reduction in negative charges on the DNA molecule, leading to reduced chromatin accessibility, and by impairing the binding efficiency of transcription factors to DNA [25]. Based on a kind of siRNA mainly measuring 24 nt in length, RdDM represents a plant-specific mechanism for the establishment and maintenance of DNA methylation, which enable the stable heritability of asymmetric CHH methylation patterns in genomic DNA [26,27,28].

**Figure 1:**
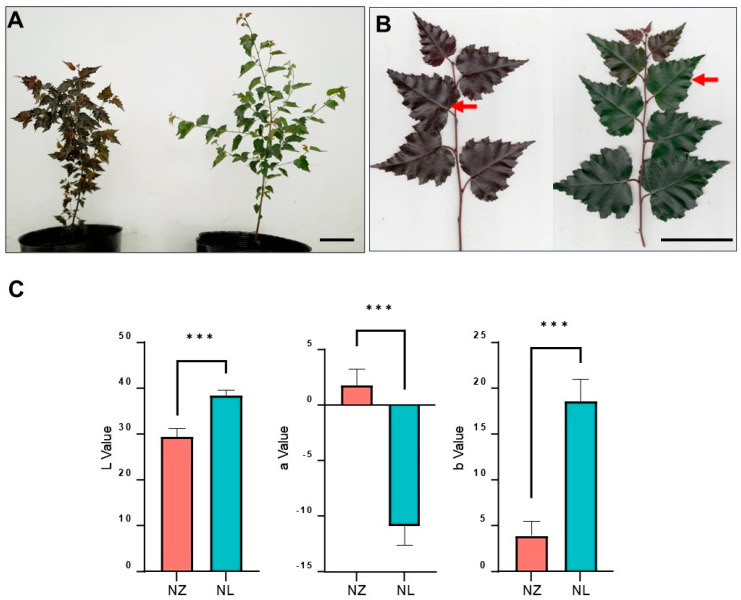
Color difference between NZ and NL. (**A**), NZ (left) and NL (right) plants propagated by tissue culturing. Bar, 10 cm. (**B**), Branch of NZ (left) and NL (right). Red arrows indicate the intermediate leaves that were collected for measurement. Bar, 5 cm. (**C**), Bar plot of leaf color trait measurement (*** = *p* < 0.0001). L represents lightness, with a positive value indicating higher brightness and a negative value indicating lower brightness; a represents the red–green saturation, with a positive value indicating a redder hue and a negative value indicating a greener hue; b represents the yellow–blue saturation, with a positive value indicating a yellower hue and a negative value indicating a bluer hue. Student’s *t*-test was used to assess the significance level between the two lines. Error bars represent standard deviation of each group.

*Betula pendula* ‘Purple Rain’ (Figure 1A–C, left), distinguished by its purple leaves [29] and notable resistance to abiotic stresses [30], holds promise for both afforestation and ornamental applications. Previous studies have identified functional genes involved in the anthocyanin biosynthesis pathway of Purple Rain birch [31]; however, the key transcription factors governing these genes remain elusive. Through tissue culture, a green mutant line NL was derived from the clone NZ of *B. pendula* ‘Purple Rain’, exhibiting diminished purple coloration in young leaves and a near-complete loss in mature ones (Figure 1A–C, right). The green leaf phenotype of NL was consistently maintained through asexual reproduction. This study employs a multi-omics sequencing approach to compare the *B. pendula* ‘Purple Rain’ clone NZ with its green mutant NL, elucidating the molecular mechanisms underlying pigment production and maintenance in variegated plants, thereby furnishing a theoretical framework for advancing genetic breeding in such plants.

## 2. Results

### 2.1. Leaf Color Phenotype Difference

The mutant NL exhibited leaf coloration that was lighter than that of the wild-type NZ during tissue culture (Supplementary Figure S1). Upon transplantation outdoors, juvenile leaves of NL displayed a weaker purple hue compared to NZ, with the purple coloration in the mature leaves nearly vanishing (Figure 1A). In June, the intermediate leaves of the mutant line NL (Figure 1B) exhibited a deep green color (L* = 38.54, a* = −10.09, b* = 18.65), contrasting with the purple tone of the *B. pendula* ‘Purple Rain’ NZ (L* = 29.57, a* = 1.84, b* = 3.96) (Figure 1C).

### 2.2. RNA-Seq and Metabolomic Analysis Showed That the Anthocyanin Biosynthesis Pathway Was Down-Regulated by Changes in Some TFs

To further determine the molecular mechanisms underlying the leaf color of *B. pendula* ‘Purple Rain’, we carried out RNA-Seq analysis. A total of 87.9 Gb of clean RNA-Seq data was acquired, with an average mapping rate to the reference *Betula* genome of 94.28%. The principal component analysis (Supplementary Figure S2A) and correlation heatmap (Supplementary Figure S2B) revealed a clear separation of gene expression profiles between the NZ and NL clones.

**Figure 2:**
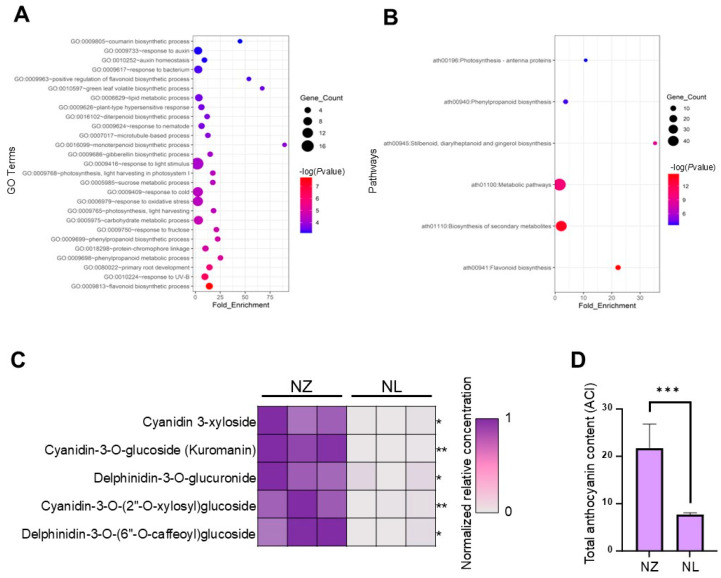
RNA-Seq and metabolomics result. (**A**), Bubble charts of GO enrichment BP terms of down-regulated DEGs. (**B**), Bubble charts of KEGG enrichment of down-regulated DEGs. (**C**), Heat map of five anthocyanin molecules’ contents with significant differences at * = *p* < 0.05, ** = *p* < 0.01. (**D**), Bar plot of total anthocyanin content in NZ and NL leaves at *** = *p* < 0.001. Student’s *t*-test was used to assess the significance level between the two lines.

In total, 18,738 genes were found to be expressed in either NZ or NL, among which 464 genes with expressions of *Padj* ≤ 0.01 and |log2FC| ≥ 1.0 were selected as differentially expressed genes (DEGs) (Supplementary Figure S2C). These DEGs were significantly enriched in thirteen pathways, including phenylalanine metabolism and flavonoid biosynthesis (Figure 2A,B). Enrichment analyses for other sets of differential genes are detailed in Supplementary Table S2. Given that the phenylalanine metabolism pathway and the flavonoid biosynthesis pathway impact anthocyanin synthesis, it is inferred that anthocyanins are the principal pigments underlying the color difference between NL and NZ. To substantiate this, targeted metabolomics using UPLC-MS/MS was employed to quantify anthocyanins in NL and NZ, revealing a significant reduction in five anthocyanins in the NL (Figure 2C). Consequently, the anthocyanin content in the NL leaves was found to be less than 40% of that in the NZ (Figure 2D).

**Figure 3:**
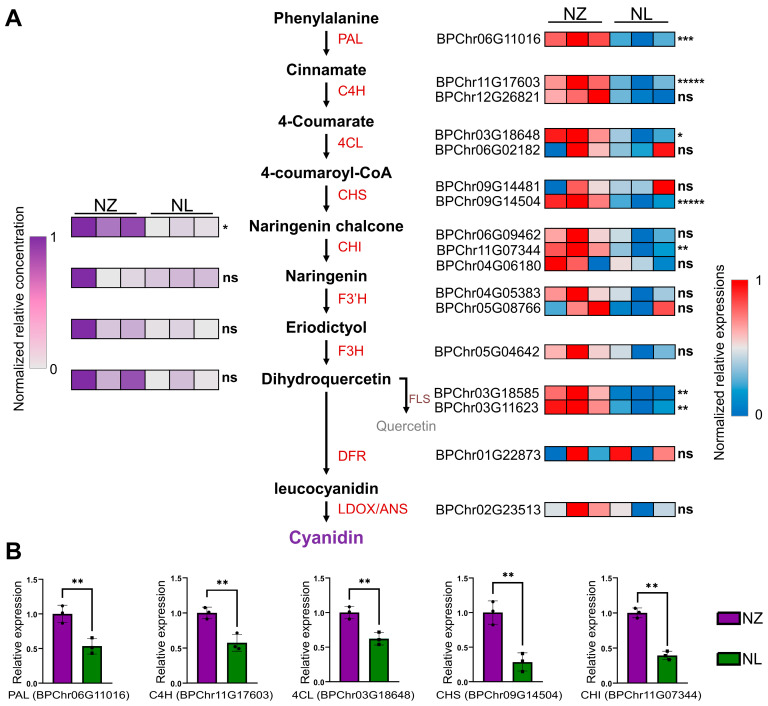
RNA-Seq and metabolomics combined analysis of anthocyanin biosynthesis pathway. (**A**), Heatmap of precursor contents for anthocyanin synthesis and anthocyanin biosynthesis structural genes expression. The left panel of heatmap represents the concentration of 4 precursor contents. The right panel of heatmap represents the expression of structural genes belonging to the nearby categories in the middle panel. Color scale represents the relative levels of metabolite concentration or relative gene expression, * = *Padj* ≤ 0.05, ** = *Padj* ≤ 0.01. *** = *Padj* ≤ 0.001, ***** = *Padj* ≤ 0.00001. ‘ns’ = no significant difference. (**B**), Bar plot of qRT-PCR result of 5 differentially expressed anthocyanin biosynthesis structural genes. ** = *p* ≤ 0.01. Student’s t-test was used to assess the significance level between the two lines. Error bars represent data range of each group. All primers are listed in Supplementary Table S1.

To elucidate the specific alterations in the anthocyanin biosynthesis process of NL, an integrative analysis was conducted, correlating gene expression profiles along the anthocyanin biosynthetic pathway with flavonoid metabolomics. The findings revealed diminished expression of *PAL*, *C4Hs*, *4CL*, *CHS*, and *CHI* (Figure 3A, right), which consequently led to reduced naringenin chalcone levels in NL (Figure 3A, left), with chalcone being a pivotal precursor for anthocyanin biosynthesis. Additional flavonoid metabolomic data are provided in Supplementary Table S3. The down-regulated transcription levels of *PAL*, *C4Hs*, *4CL*, *CHS*, and *CHI* in NL were further validated by qRT-PCR (Figure 3B). In the NL, a decrease in the transcription of genes involved in chalcone synthesis within the phenylalanine metabolic pathway was observed, which indicates that some of the transcription factors that could activate this pathway have a reduction or destruction of function, or that an abnormal function enhancement exists in the transcription repressor factors that down-regulate this pathway. A WGS was then deployed to reveal this variated transcription factor.

### 2.3. SNPs/InDels Mutations Are Not Supported as a Cause of Phenotypic Variation

To explore the potential genetic variations regarding the leaf color changes, we performed WGS in the NZ and NL. A total of 131 Gbp of raw WGS data was obtained from NZ and NL, with Q30 scores exceeding 90%. For the sake of the rigor of the SNP analysis, only reads for high-quality alignment (screened by SAMtools with ‘-q’ parameter = 20) were retained. Among these high-quality reads, SNP calling was performed with only pair ends that were perfectly aligned to the genome with a positive and negative pair (screened by SAMtools with ‘-f’ parameter = 2). These reads, which are of high quality and correctly aligned, account for 75.26% of the total data and yielded 304,215,255 pairs of high-quality reads, from which 10,211,111 SNPs/InDels markers were retained from NZ and NL. A principal component analysis (PCA), incorporating SNP data from five green-leaf wild-type *B. pendula*, indicated that NL and NZ belong to the same clonal lineage (Supplementary Figure S3). A total of 7552 mutations with distinct genotypes were identified, comprising 139 non-synonymous mutations affecting 127 genes. According to the annotation results (Supplementary Table S4), none of these variant genes were recognized as transcription factors influencing anthocyanin accumulation genes *PAL*, *C4Hs*, *4CL*, *CHS*, and *CHI*, at the same time as the result of the RNA-Seq. Although a non-synonymous mutation was found in *BPChr06G11016* (*PAL2*), the diminished ability to synthesize anthocyanins should not be attributed to variation in this single gene, otherwise the reduction in other functional genes cannot be explained. SNPs/InDels mutations are not thought to be responsible for the leaf color changes in *B. pendula* ‘Purple Rain’ during tissue cultures.

### 2.4. Epigenetic Analysis Reveals Regulation of Anthocyanin Biosynthesis by MYB113

**Figure 4:**
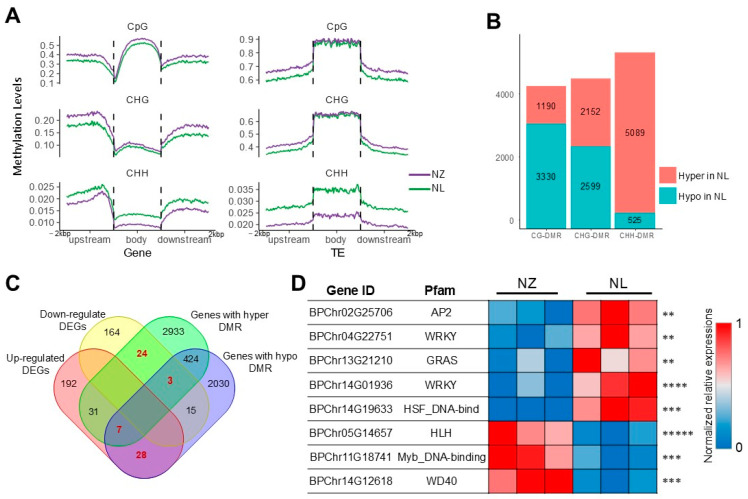
WGBS and RNA-Seq combined analysis detected TFs affected by epigenetic regulation. (**A**), DNA methylation level by context around genes and transposon regions. Purple curves represent methylation level of NZ; green curves represent methylation level of NL. (**B**), DMR numbers. (**C**), Venn plot showing DEGs carrying DMRs. (**D**), Differentially expressed TFs carrying DMRs, ** = *Padj* ≤ 0.01, *** = *Padj* ≤ 0.001, **** = *Padj* ≤ 0.0001, ***** = *Padj* ≤ 0.00001.

DNA methylation, a prominent epigenetic modification, frequently leads to the repression of gene expression in the vicinity of hypermethylated regions. To investigate the epigenetic modifications underlying the green leaf phenotype in the NL lineage, comprehensive genome-wide DNA methylation profiles of NL and NZ were examined. In total, 71.1 Gbp of clean WGBS data with an unmethylated cytosine BS conversion (C to T) rate over 99% (according to the report on the quality of sequencing data provided by Annoroda Gene Technology) were generated, and the average alignment rate to the reference *Betula* genome was 91.4%. The DNA methylation at the genome-wide level is shown in Supplementary Figure S4. The analysis of the methylation differences revealed elevated CHH methylation levels in the NL compared to the NZ, notably in both genic regions and transposable elements (TEs) regions (Figure 4A). The differential methylation site analysis disclosed that while the majority of the differing CG sites showed hypomethylation status in the NL, a predominant trend of hypermethylation at differential CHH sites was observed in the NL (Figure 4B).

CHH methylation in higher plants is often maintained through the RdDM pathway [26,27,28], relying on 24 nt small RNAs, thereby exhibiting a tight correlation with small RNA abundance. Consequently, small RNA sequencing was performed on NL and NZ samples. This yielded 50.06 million clean small RNA reads, with an average mapping rate of 90.57%. The combined small RNA datasets from the NL and NZ were assembled and compared against previously reported small RNA sequencing data for European white birch [32], yielding specific small RNA precursors for *B. pendula* ‘Purple Rain’, detailed in Supplementary Table S5.

A comparative assessment of the abundance of 21–22 nt small RNAs across various gene loci in NL and NZ revealed discrepancies affecting 240 genes (expression *Padj* ≤ 0.01 and |log2FC| ≥ 1.0) (Supplementary Figure S5A). However, these differential small RNAs led to transcript abundance variations in only two genes (Supplementary Figure S5B), both of which, upon functional annotation, were found to be unrelated to anthocyanin biosynthesis (*BPChr14G20643*, serine hydrolase (FSH1), EF-hand_1, FSH1) (*BPChr06G16470*, NB-ARC, RPW8).

In contrast, the impact of differentially methylated sites on gene expression variability is more profound. A total of 62 differential genes exhibit differential methylation modifications (Figure 4C), predominantly characterized by elevated transcription levels accompanying reduced methylation (28 cases) and, conversely, decreased transcription accompanying increased methylation (24 cases). To elucidate the key epigenetic variants underlying the differential expression of the phenylalanine metabolic pathway, eight transcription factors among the 62 differentially methylated genes were annotated (Figure 4D), implicating families such as *AP2*, *WRKY*, *GRAS*, *HLH*, *HSF*, *MYB*, and *WD40*. While members of the *AP2*, *WRKY*, and *WD40* families are generally implicated in a potential positive correlation with anthocyanin synthesis, according to the eggNOG-mapper annotation (Supplementary Table S6), the most direct association is observed with *BPChr11G18741*, a gene belonging to the Myb_DNA-binding family. Based on alignment outcomes, this gene has been designated as *BpMYB113*.

**Figure 5:**
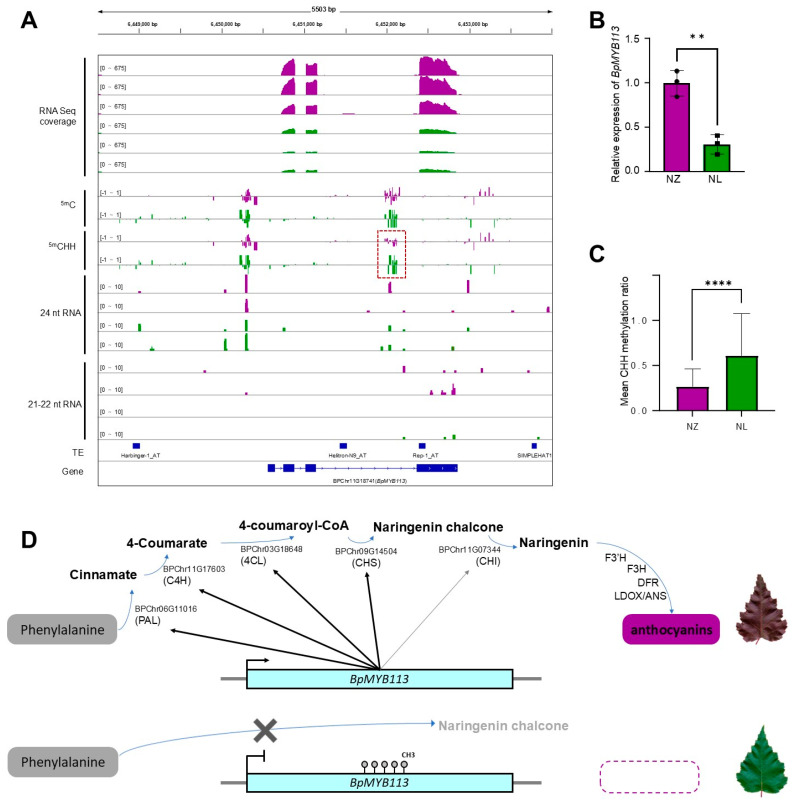
Epigenetic mutation of *BPChr11G18741* (*BpMYB113*) regulates anthocyanin biosynthesis. (**A**), Gene expression level, DNA methylation level, and small RNA abundance of *BPChr11G18741* (*BpMYB113*). Purple plots represent NZ and green plots represent NL. Red square represents DMR by CHH context. (**B**), Bar plot of qRT-PCR result of *BPChr11G18741* (*BpMYB113*), ** = *p* ≤ 0.01. Student’s *t*-test was used to assess the significance level between the two lines. Error bars represent data range of each group. (**C**), Mean CHH methylation ratios of DMR. **** = *p* ≤ 0.0001. Mann–Whitney U-test was used to assess the significance level between the two lines. Error bars represent standard error. (**D**), Mechanism of leaf color changes in NL. The low expression of *BpMYB113* associated with DNA methylation weakens the transcription of some genes required for anthocyanin synthesis. × represents the weakening of metabolic pathways; purple dotted square represents the absence of anthocyanin.

*BpMYB113* carries a hyper CHH methylation site in NL (Figure 5A), coinciding with a down-regulated expression level (Figure 5B). This differential CHH methylation site, situated within the gene body, is significantly hyper in NL (Figure 5C). Although the CHH methylation site is accompanied by the presence of some 24 nt siRNAs (Figure 5A), no differential content of 21–22 nt or 24 nt RNAs was discerned between NL and NZ. The low expression of *BpMYB113* directly or indirectly influenced the transcript level of *BPChr06G11016* (*PAL*), *BPChr11G17603* (*C4H*), *BPChr03G18648* (*4CL*), *BPChr09G14504* (*CHS*) and *BPChr11G07344* (*CHI*). The decreased expression of these genes led to a decrease in flavonoids, especially naringenin chalcone, the most important substrate for anthocyanin synthesis, and eventually caused a green leaf color (Figure 5D).

## 3. Materials and Methods

### 3.1. Plant Materials

A wild-type line of *B. pendula* ‘Purple Rain’ (NZ) is distinguished by its purple leaves and cultivated by vegetative propagation (Figure 1A–C, left) through a previously described tissue culture protocol [33]. A green-leaf mutant line (NL) is from NZ (Figure 1A–C, right). One-year-old seedlings of NZ and NL grew in plastic pots at birch seed orchard (126.622615 east longitude, 45.716848 north latitude) in Northeast Forestry University. Soil substrate, growth conditions, and field management were established following previous cultivation experience [30]. For leaf color trait measurement, one leaf was collected from each of the 3 seedlings of each line. For flavonoids-targeted metabolomics determination, RNA sequencing, and qRT-PCR, 3 to 5 leaves were collected from each of the 3 seedlings of each line as 3 biological replicates. For small RNA sequencing, 3 to 5 leaves were collected from each of the 2 seedlings of each line as 2 biological replicates. For WGS, 3 to 5 leaves were collected from each of the 2 seedlings of NZ line and 3 seedlings of NL line as 2 or 3 biological replicates. For WGBS, 1 leaf was collected from each of 15 seedlings of each line and mixed as 2 pools. The leaf samples for sequencing and metabolomics determination were quickly frozen with dry ice after they were collected. The leaf samples for qRT-PCR were quickly frozen with liquid nitrogen after they were collected. All leaf materials in this study were intermediate leaves of a branch (Figure 1C) and collected on 1 June 2022.

### 3.2. Leaf Color Measurement

Leaf colors were quantified into the L*, a*, b* color space (CIE 1976, CIELAB) by color difference meter (CR-400, Minolta, Osaka, Japan). The L value indicates the brightness of the color and can range from 0 to 100, where 0 is pure black and 100 is pure white. In color measurement, the L value determines the brightness and contrast of an object’s surface, and thus evaluates its appearance quality. The A value represents the red–green color, and the value ranges from −128 to +127, where −128 is green and +127 is red. By measuring the a-value of an object’s surface, it is possible to know whether the surface of an object is biased to red or green, so as to evaluate its color accuracy and consistency. a > 0 is red and a < 0 is green. The b-value represents the yellow–blue degree of the color and ranges from −128 to +127, where −128 is blue and +127 is yellow. By measuring the b-value of an object’s surface, it is possible to know whether the surface of an object is biased to yellow or blue, so as to evaluate its color accuracy and consistency. b > 0 is yellow and b < 0 is blue. Leaf anthocyanin content was estimated by anthocyanin content tester (OPTI-SCIENCES ACM-200+, Hudson, NH, USA).

### 3.3. Flavonoids-Targeted Metabolomics Determination

Three biological repetitions were performed for NL and NZ. Metabolite extraction and determination were performed by Metware Biotechnology (Wuhan, China) with SHIMADZU Nexera X2 (Kyoto, Japan) and Applied Biosystems 4500 QTRAP platform (Fremont, CA, USA). Significantly regulated metabolites between groups were determined by VIP ≥ 1 and absolute of base-2 logarithm of fold change (|log2FC|) ≥ 1, from which VIP values were extracted from OPLS-DA results and generated with R package MetaboAnalystR 3.0 [34,35,36].

### 3.4. Whole Genome Re-Sequencing (WGS)

DNA extraction, database building, sequencing, data quality control, and data cleaning were completed by Annoroda Gene Technology (Beijing, China). The Illumina Novaseq6000-Sequencing platform (San Diego, CA, USA) was used. Clean sequencing data from multiple times of database building were merged to ensure the final sequencing depth for each line was over 50×. Clean reads were aligned to reference *Betula* genome [37] using BWA (Version: 0.7.18) [38]. Processing files were parsed using SAMtools (Version: 1.20) [39], and mutations including single-nucleotide polymorphisms (SNPs) and small insertions and deletions (InDels) were identified using BCFtools (Version: 1.20) [40]. Different SNPs/InDels were screened with Fisher test, with *p* ≤ 0.01 as the threshold, and the effects were predicted with AnnoSNP (https://github.com/lhui2010/AnnoSNP, accessed on 19 October 2024). Variant genes were annotated with eggNOG-mapper (Version: 2.1.12) [41]. Principal components of SNPs were analyzed with Plink (Version: 1.90) [42]. WGS data of five wild-type *B. pendula* randomly selected form birch seed orchard at Northeast Forestry University were sequenced using the same sequencing platform as in some of our previous studies.

### 3.5. RNA Sequencing

Three biological repetitions were performed for each line. DNA extraction, database building, sequencing, and data quality control were completed by Annoroda Gene Technology (Beijing, China) using the Illumina Novaseq6000-Sequencing platform, with sequencing data over 6 GbP for each replicate. Clean reads were aligned to reference *Betula* genome [37] using Hisat2 (Version 2.2.1) [43]. Gene expressions were quantified using StringTie (Version: 2.2.3) [44,45]. Gene expression difference analyses were performed using the R package DESeq2 (https://bioconductor.org/packages/release/bioc/html/DESeq2.html, accessed on 19 October 2024) [46]. Genes with expressions of *Padj* ≤ 0.01 and |log2FC| ≥ 1.0 were considered differentially expressed genes (DEGs). KEGG enrichment analyses were performed using The Database for Annotation, Visualization and Integrated Discovery (DAVID, Version: v2023q4) [47].

### 3.6. Small RNA Sequencing

Two biological repetitions were performed for each line. RNA extraction, library construction, and sequencing were completed by Biozeron company (Shanghai, China) using the Illumina Novaseq6000 Sequencing platform with sequencing data of 10 M reads for each replicate. Data quality control, filtering, and adapter removal were performed using fastp (Version: 0.23.4) [48]. Small RNA de novo predictions were performed using miRDeep-P2 (https://github.com/TF-Chan-Lab/miRDeep-P2_pipeline, accessed on 19 October 2024) [49]. In total, 21–22 nt micro-RNA reads were aligned to the reference *Betula* genome [37] using bowtie2 (Version: 2.5.4) [50] and counted using HTSeq-count (Version: 2.0.4) [51]. Significant differences of 21–22 nt micro-RNA numbers on each gene were analyzed using independent samples *t*-test. Genes with 21–22 nt RNA numbers of *Padj* ≤ 0.01 and |log2FC| ≥ 1.0 were considered differentially mi-RNA-regulated genes.

### 3.7. Whole Genome Bisulfite Sequencing (WGBS)

Total DNA was extracted using a DNA Extraction Kit (bio-filtration column type; KONVIER Group, Beijing, China). DNA bisulfite treatment, database building, sequencing, and data quality control were completed by Annoroda Gene Technology (Beijing, China) using the Illumina Novaseq6000-Sequencing platform with a sequencing depth for each line over 20×. The acquired sequence data were aligned to the reference *Betula* genome [37] and the methylation level was analyzed using bsmap (Version 2.90) [52].

### 3.8. qRT-PCR

Total RNA was extracted using a plant RNA Extraction Kit (BioTeke Co., Beijing, China) and reverse-transcribed to cDNA using a PrimeScript RT reagent kit with gDNA Eraser (Takara, Osaka, Japan). The reverse-transcribed cDNA at 1:10 served as a template. The SYBR Green PCR kit (Toyobo Co., Ltd., Osaka, Japan) was used for qRT-PCR amplification. In addition, 18S RNA was used as the internal reference gene, like in the previous study on *B. pendula* ’Purple Rain’ [30].

The relative expression of each gene was calculated using the formula RE = 2^−ΔΔCt^, where CT is the cycle threshold, representing the number of cycles required for the fluorescent signal in the reaction tube to reach the threshold, ΔCt = the CT value of the target gene − the average CT value of the internal reference gene, and ΔΔCt = ΔCT (sample 1) − ΔCT (sample 2), and the gene expression in NZ leaf was used as a control. All primers are listed in Supplementary Table S1.

## 4. Discussion

The green mutant NL of *B. pendula* ‘Purple Rain’, when cultivated under field conditions, exhibits pale purple juvenile leaves transitioning to green in maturity (Figure 1A–C), contrasting with the uniformly purple foliage of *B. pendula* ‘Purple Rain’ and the consistently green leaves of European white birch. This unique phenotype enables the creation of more visually diverse horticultural landscapes. NL, with its reduced anthocyanin content, serves as a valuable material for studying the biosynthesis of birch anthocyanins, facilitating comparisons with *B. pendula* ‘Purple Rain’ to uncover additional factors influencing total anthocyanin accumulation. In prior comparative studies between *B. pendula* ‘Purple Rain’ and European white birch, chalcone synthase was identified as a pivotal gene responsible for the purple pigmentation in *B. pendula* ‘Purple Rain’ [31]. By integrating flavonoid metabolomics with transcriptomic analyses, our study uncovered a concurrent transcriptional down-regulation of the entire phenylalanine metabolic pathway, including chalcone synthase, in NL, leading to a reduction in total chalcone levels, whereas genes in the anthocyanin biosynthetic pathway showed no substantial alterations (Figure 3A). The variations observed in NL predominantly affect phenylalanine metabolism, evidenced by significant down-regulation of four phenylalanine metabolic genes and one CHI gene. However, there is no indication that these mutations exert an impact on *BpChr05G08766* (encoding *F3*′H) or *BPChr01G22873* (encoding *DFR*).

In the tissue culture system utilized for establishing and propagating the NL line, cells underwent dedifferentiation to form callus tissue, which subsequently re-differentiated into plantlets [33]. During the process of dedifferentiation in plant cells, which enables the restoration of totipotency through reprogramming, alterations in methylation levels occur, rendering the cells susceptible to epigenetic variations at random sites [53,54].

Concurrently, spontaneous occurrences of random DNA sequence mutations also take place. Both genetic mutations and epigenetic variations must be considered in mutant studies, and the epigenetic nature of traits cannot be definitively ascertained until recurrent instances of mutants spontaneously reverting to the wild type are observed. This investigation employed WGS to rule out genetic mutations before ultimately characterizing the epigenetic alterations in NL. NL exhibits genome-wide increases in CHH methylation in both genic regions and transposable element regions, suggesting activation or reinforcement of the de novo establishment or maintenance mechanisms of CHH methylation. This phenomenon may be associated with hormonal stimuli [55] or osmotic stress experienced during tissue culture processes [56].

MYB proteins are characterized by their highly conserved MYB-repeat domains, whose binding specificity and interacting partners dictate their precise regulatory effects on target genes [15]. *MYB113* has been demonstrated in various tree species to play a positive regulatory role in anthocyanin biosynthesis, to the extent that artificially elevating its transcription level has led to the creation of new tree varieties with purple organs [16,17,18]. In this study, *BpMYB113* stands as the sole transcription factor in NL causing an epigenetic trait heritable during asexual reproduction, with a known positive regulatory function in anthocyanin synthesis. Its hyper methylation site within the gene body is correlated with reduced transcriptional activity. The expression of *BpMYB113* exhibits spatial and temporal dynamics; its down-regulation does not completely abolish the expression of all phenylalanine metabolic and anthocyanin biosynthesis genes, allowing for the retention of anthocyanins in NL’s young leaves, manifesting as a purple hue.

DNA methylation frequently influences anthocyanin accumulation in plants. In *Raphanus sativus* ‘Xinlimei’, a CACTA transposon situated within the promoter region of the *RsMYB1* gene acts as a transcriptional enhancer, leading to substantial anthocyanin accumulation in roots [57]. Upon methylation of this CACTA transposon, reduced transcription of *RsMYB1* results in the disappearance of anthocyanins in the roots [57]. The transcription factor *CmMYB6* in *Chrysanthemum morifolium* regulates anthocyanin accumulation in YP petal tissues. Individuals with methylated *CmMYB6* promoters exhibit a shift from pink to yellow petals due to decreased anthocyanins; conversely, targeted demethylation of the promoter restores the pink petal color [6]. *XsMYB113*, which governs the basal petal color in *Xanthoceras sorbifolium*, experiences differential transcription levels at various flower developmental stages due to altered promoter methylation patterns, causing a gradual color change in the petal base. Furthermore, target genes in the anthocyanin biosynthesis pathway under MYB regulation also exhibit transcript level variations due to methylation modifications; for instance, the uneven distribution of methylation on *PrDFR* and *PrANS* genes in *Paeonia rockii* ‘Xibei’ petals contributes to disparities in anthocyanin content, giving rise to petal spotting [58]. In this instance, the differential methylation site of *BpMYB113* occurs in the CHH context, a feature that is asymmetric in double-stranded DNA molecules and is peculiar to plants. Both the de novo establishment and maintenance of CHH methylation are associated with the RNA-directed DNA methylation (RdDM) mechanism [26,27]. The analysis of the microRNAs revealed the presence of 24 nt RNA at the differential CHH methylation site of the key variant gene, *BpMYB113*, in the NL (Figure 5A). However, no substantial increase in 24 nt RNA levels was observed concomitantly with the heightened methylation at this site in NL, indicating that the underlying mechanism driving this variation remains to be elucidated.

Gene body methylation (gbM) refers to genes with an enrichment of CG DNA methylation within the transcribed regions and depletion at the transcriptional start and termination sites [59]. The function of gbM remains elusive; gbM genes were expressed, on average, in more single-cell replicates than unmethylated genes [60], which means that gbM has some positive influence on transcription. However, at the common CG/CHG gbM site in *BpMYB113*, cytosine in the CHH context was methylated in the NL line (Figure 5A). This modification might change the original function of this gbM site and enhance the negative effects of DNA methylation on chromatin openness by switching the mean methylation into an over-hyper level [25,57], and therefore reducing the expression of *BpMYB113*. Demethylation editing with dCas9-TET tools targeting *BpMYB113* in both *B. pendula* ‘PurpleRain’ and the green mutant NL will be carried out in our future study to explore the role of this atypical gbM site in the gene regulation of birch.

## 5. Conclusions

The mutant NL exhibited leaf coloration that was lighter than that of the wild-type *B. pendula* ‘Purple Rain’. RNA-Seq and metabolomic analysis revealed that the changing of the NL leaf color was due to a lower anthocyanin concentration, caused by the reduced expression of multiple functional genes. Genetic variations are not supported as a cause of these changes in NL, which suggests that the phenomenon is a result of epigenetic regulation. A multi-omics analysis of WGBS, small RNA-Seq, and RNA-Seq designated the key gene as *BpMYB113*, which carries a hyper CHH methylation site in NL. This methylation site is associated with a lower expression of *BpMYB113*, which weakens the transcription of genes required for anthocyanin synthesis.

## Data Availability

All high-throughput sequencing data have been uploaded and published in the Bio-Project PRJCA030863 of China National Center for Bioinformatics (https://ngdc.cncb.ac.cn/bioproject/browse/PRJCA030863, accessed on 19 October 2024).

## Supplementary Materials

The following supporting information can be downloaded at: https://www.mdpi.com/article/10.3390/ijms252212030/s1.
