# Supplementary figures and images for: Epigenetic Regulation of Anthocyanin Biosynthesis in Betula pendula ‘Purple Rain’
Source: Int J Mol Sci. 2024 Nov 8;25(22):12030. doi: 10.3390/ijms252212030 (PMC11593655; doi:10.3390/ijms252212030)

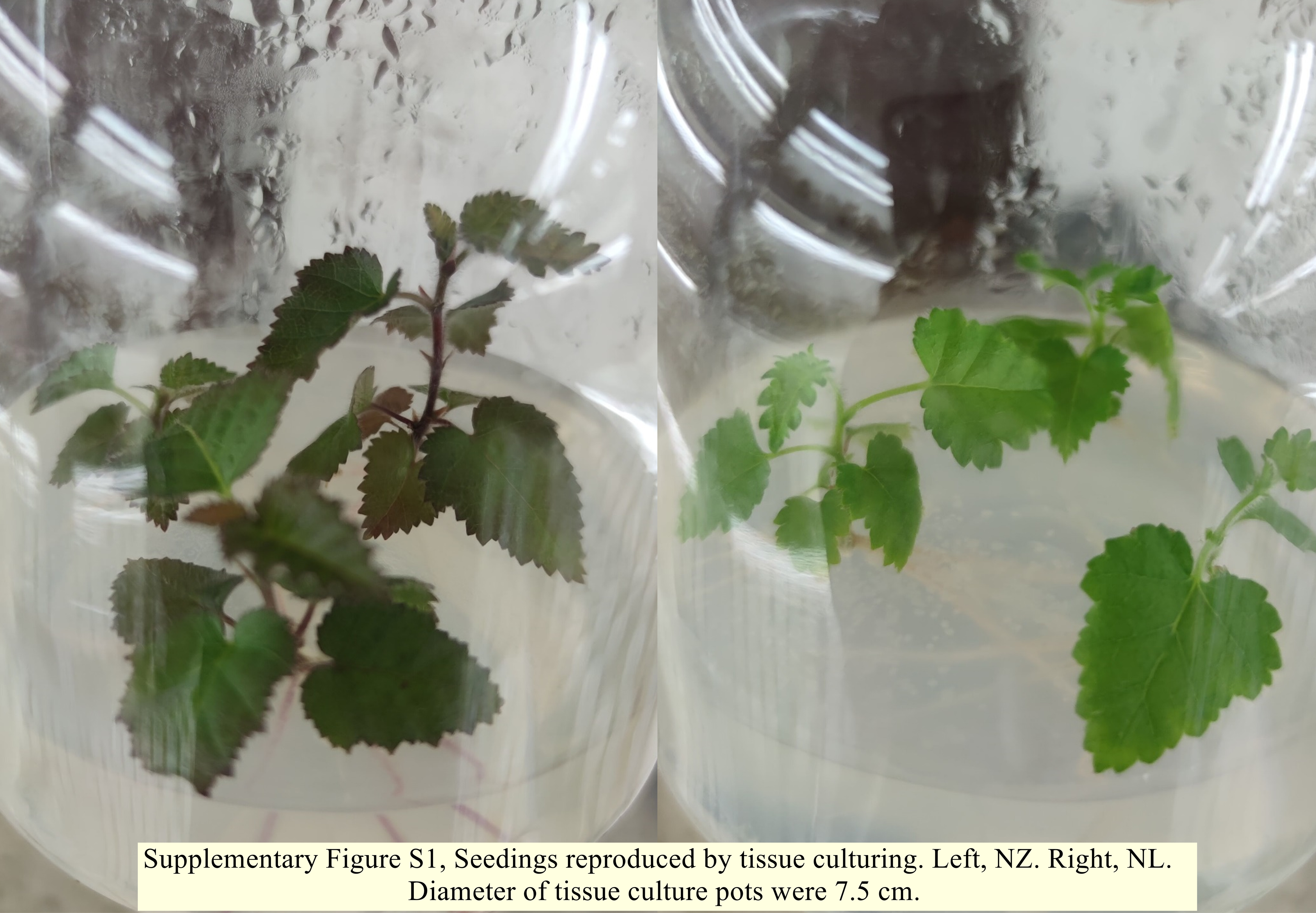

Supplement: Supplementary file 1 [file ijms-25-12030-s001.zip › Supplementary Figure S1.png]

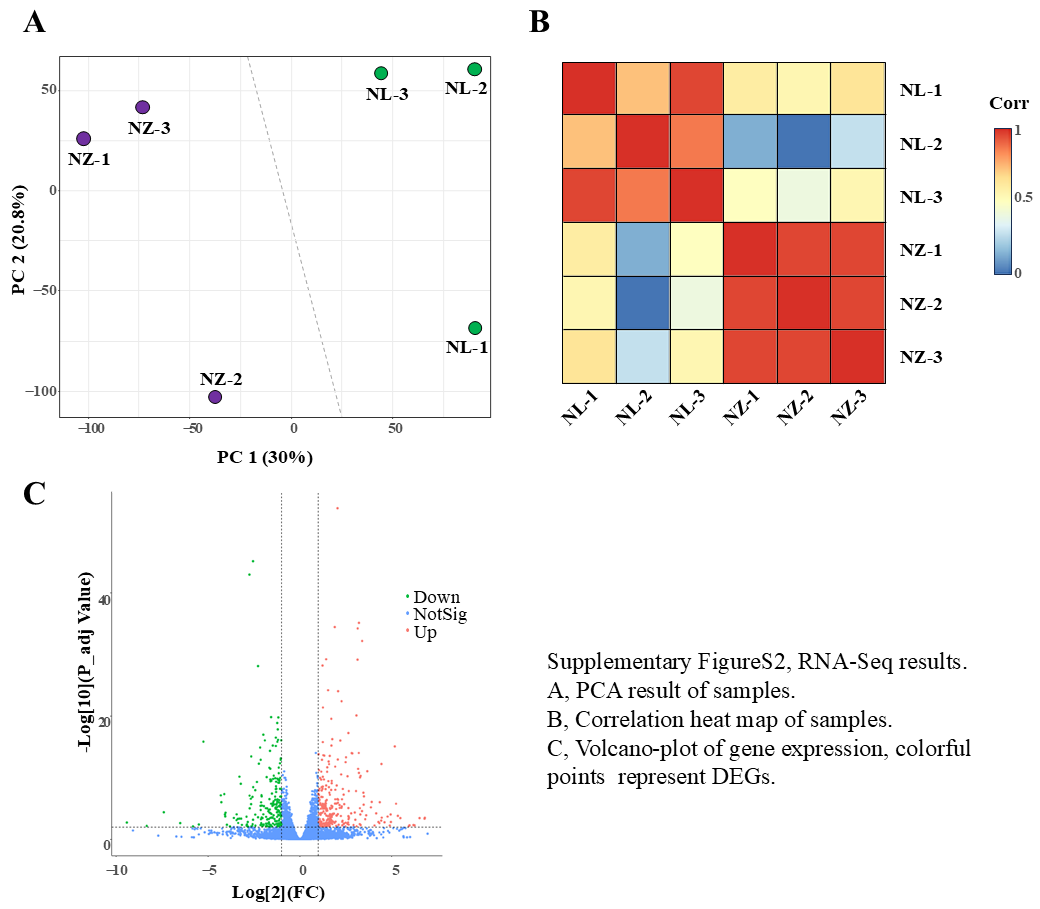

Supplement: Supplementary file 1 [file ijms-25-12030-s001.zip › Supplementary Figure S2.png]

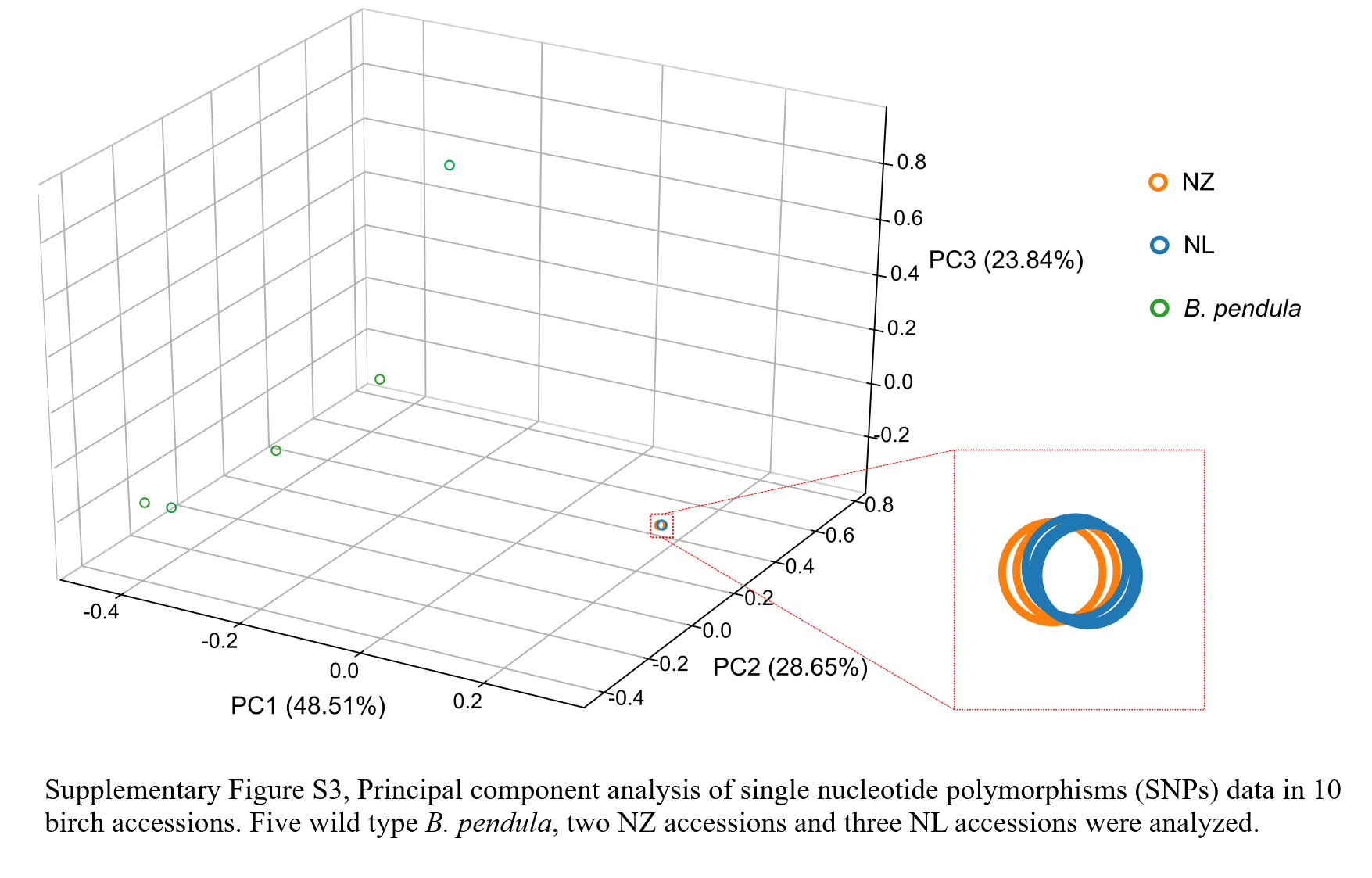

Supplement: Supplementary file 1 [file ijms-25-12030-s001.zip › Supplementary Figure S3.png]

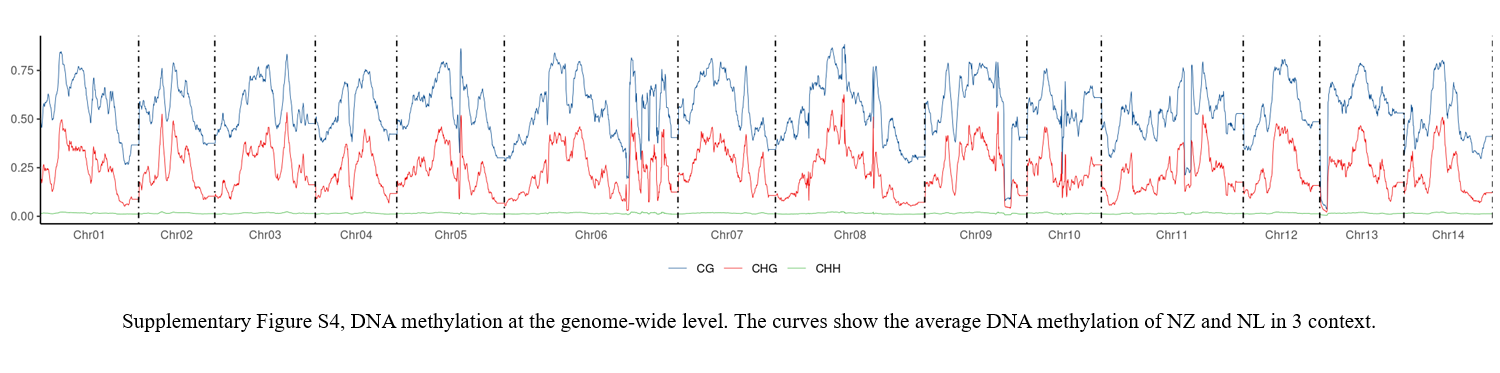

Supplement: Supplementary file 1 [file ijms-25-12030-s001.zip › Supplementary Figure S4.png]

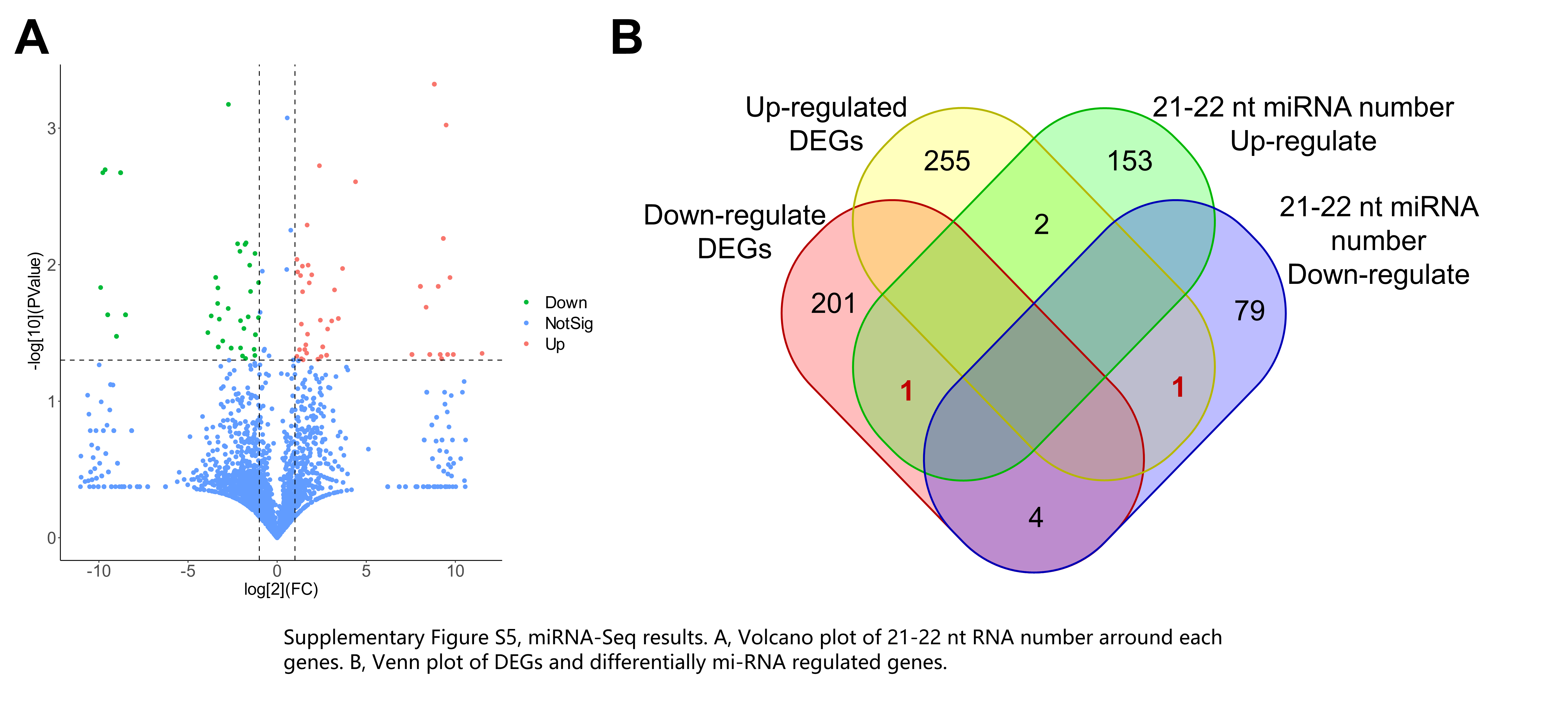

Supplement: Supplementary file 1 [file ijms-25-12030-s001.zip › Supplementary Figure S5.png]
